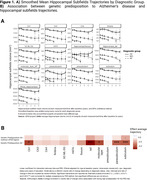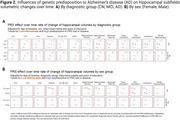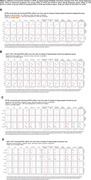# Exploring sex‐specific accelerated hippocampal atrophy across AD stages beyond *APOE‐ε4* assessment in genetic risk evaluation

**DOI:** 10.1002/alz.094595

**Published:** 2025-01-09

**Authors:** Tavia E Evans, Natalia Vilor‐Tejedor, Albert Rodrigo, Patricia Genius, Blanca Rodríguez‐Fernández, Federica Anastasi, Arcadi Navarro, Hieab H.H. Adams, Juan Domingo Gispert

**Affiliations:** ^1^ Erasmus MC, Netherlands, Rotterdam Netherlands; ^2^ Barcelonaβeta Brain Research Center (BBRC), Pasqual Maragall Foundation, Barcelona Spain; ^3^ Radboud University Medical Center, Nijmegen, Gelderland Netherlands; ^4^ Barcelonaβeta Brain Research Center (BBRC), Pasqual Maragall Foundation,, Barcelona Spain

## Abstract

**Background:**

Current evidence suggests that hippocampal subfields have partially different genetic architecture and may improve the sensitivity of the detection of Alzheimer’s disease (AD). In this study, we investigated whether genetic predisposition to AD contributes to the accelerated rate of hippocampal volume atrophy across sex and AD stages and how this contribution is specifically driven by functional variants located in the *APOE* gene.

**Methods:**

The study comprised 1,051 participants from ADNI cohort (75.2 yo; 42.3% women), with complete demographic, genetic, and magnetic resonance imaging scans at baseline and every 6 months for a total of 96 months (Controls (amyloid negative), CUN = 363; mild cognitive impairment, MCIN = 474, AD patients, ADN = 214). Hippocampal subfields were extracted using the longitudinal processing method within FreeSurfer (v6.0). Genetic predisposition to AD was assessed through polygenic scoring with and without considering the *APOE* región. Effects of polygenicity of plasma *APOE*, functional *APOE* brain eQTLs and CSF pQTL were also considered. Linear mixed‐effect models with random‐ time slope and intercept for individuals were used to investigate the association between genetic predisposition to AD and hippocampal subfields volumetric trajectories over time. Models were adjusted by age, sex, AD disease status, years of education and total hippocampal volume. Disease and sex status‐specific trajectories dependent on genetics predisposition were assessed by including an interaction term.

**Results:**

Significant hippocampal subfield volume reductions were observed, with more pronounced atrophy in MCI and AD participants [**Figure 1A]**. High genetic predisposition to AD was associated with accelerated atrophy in various subfields [**Figure 1B]**. MCI individuals with high genetic predisposition showed more severe atrophy in several subfields, with sex‐related differences observed, particularly in women [**Figure 2**]. Notably, these effects were non‐significant when excluding *APOE* variants. Further functional analyses pinpointed CSF pQTLS of the A*POE* gene and *APOE*‐e4 carriership status as primary drivers [**Figure 3**].

**Conclusions:**

Our findings underscore the significance of genetic predisposition, especially functional *APOE* variants, in understanding AD progression and sex‐specific trajectories. The results reveal varying atrophy patterns across disease stages, offering crucial insights for refining AD detection and tailoring interventions for more effective management.